# Skeletal Muscle, but not Cardiovascular Function, Is Altered in a Mouse Model of Autosomal Recessive Hypophosphatemic Rickets

**DOI:** 10.3389/fphys.2016.00173

**Published:** 2016-05-13

**Authors:** Michael J. Wacker, Chad D. Touchberry, Neerupma Silswal, Leticia Brotto, Chris J. Elmore, Lynda F. Bonewald, Jon Andresen, Marco Brotto

**Affiliations:** ^1^Muscle Biology Research Group, School of Medicine, University of Missouri-Kansas CityKansas City, MO, USA; ^2^School of Health Studies, University of MemphisMemphis, TN, USA; ^3^Bone-Muscle Collaborative Science, College of Nursing and Health Innovation, University of Texas at ArlingtonArlington, TX, USA; ^4^Bone Biology Research Group, School of Dentistry, University of Missouri-Kansas CityKansas City, MO, USA

**Keywords:** osteomalacia, *DMP1*, Autosomal recessive hypophosphatemic rickets, bone-muscle crosstalk, sarcopenia, cardiovascular disease, skeletal muscle, cardiac muscle

## Abstract

Autosomal recessive hypophosphatemic rickets (ARHR) is a heritable disorder characterized by hypophosphatemia, osteomalacia, and poor bone development. ARHR results from inactivating mutations in the *DMP1* gene with the human phenotype being recapitulated in the *Dmp1* null mouse model which displays elevated plasma fibroblast growth factor 23. While the bone phenotype has been well-characterized, it is not known what effects ARHR may also have on skeletal, cardiac, or vascular smooth muscle function, which is critical to understand in order to treat patients suffering from this condition. In this study, the extensor digitorum longus (EDL-fast-twitch muscle), soleus (SOL–slow-twitch muscle), heart, and aorta were removed from *Dmp1* null mice and *ex-vivo* functional tests were simultaneously performed in collaboration by three different laboratories. *Dmp1* null EDL and SOL muscles produced less force than wildtype muscles after normalization for physiological cross sectional area of the muscles. Both EDL and SOL muscles from *Dmp1* null mice also produced less force after the addition of caffeine (which releases calcium from the sarcoplasmic reticulum) which may indicate problems in excitation contraction coupling in these mice. While the body weights of the *Dmp1* null were smaller than wildtype, the heart weight to body weight ratio was higher. However, there were no differences in pathological hypertrophic gene expression compared to wildtype and maximal force of contraction was not different indicating that there may not be cardiac pathology under the tested conditions. We did observe a decrease in the rate of force development generated by cardiac muscle in the *Dmp1* null which may be related to some of the deficits observed in skeletal muscle. There were no differences observed in aortic contractions induced by PGF_2α_ or 5-HT or in endothelium-mediated acetylcholine-induced relaxations or endothelium-independent sodium nitroprusside-induced relaxations. In summary, these results indicate that there are deficiencies in both fast twitch and slow twitch muscle fiber type contractions in this model of ARHR, while there was less of a phenotype observed in cardiac muscle, and no differences observed in aortic function. These results may help explain skeletal muscle weakness reported by some patients with osteomalacia and need to be further investigated.

## Introduction

Hypophosphatemic rickets is caused by a group of genetic disorders characterized by reduced renal phosphate transport, bone malformation, and elevated fibroblast growth factor 23 (FGF23). FGF23 is primarily released by osteocytes and osteoblasts and regulates serum phosphate levels by increasing phosphate excretion in the kidney via regulation of sodium-phosphate transporters as well as by reducing 1,25 (OH)_2_D levels (Shimada et al., 2004; Berndt and Kumar, 2007). The most common of these genetic disorders is X-linked hypophosphatemia (XLH), followed by Autosomal Recessive Hypophosphatemic Rickets (ARHR), Autosomal Dominant Hypophosphatemic Rickets (ADHR), tumor-induced osteomalacia (TIO), and others. ARHR1 (OMIM #241520) is caused by mutations in Dentin Matrix Protein 1, DMP1 (Feng et al., 2006) whereas ARHR2 is caused by mutations in ectonucleotide phyrophospatase/phosphodiesterase 1, ENPP1 (Lorenz-Depiereux et al., 2010). ARHR due to the DMP1 mutation is characterized by hypophosphatemia, osteomalacia, and poor bone and tooth development and structure and we have previously shown that FGF23 is elevated in osteocytes (Feng et al., 2006; Farrow et al., 2009) similar to what occurs in XLH (Liu et al., 2007; White et al., 2014).

DMP1 is considered to be an extracellular matrix protein that belongs to the SIBLING (small integrin-binding ligand, N-linked glycoprotein) family and regulates biomineralization. The mechanisms responsible for DMP1-mediated bone changes still remain to be completely elucidated. However, DMP1 is thought to participate in mineralization via its role in extracellular matrix formation, increase in Wnt/β-catenin, decrease in expression of DKK1, an inhibitor of the Wnt/β-catenin signaling pathway, and inhibition of FGF23 expression (Lu et al., 2011; Feng et al., 2013; White et al., 2014). In regards to FGF23 expression, it is hypothesized that DMP1, together with PHEX (phosphate-regulating gene with homologies to endopeptidases on the X chromosome which is mutated in XLH), regulates the expression/release of FGF23 by osteocytes as well as the degradation of FGF23 (Martin et al., 2011, 2012; Lee et al., 2014). It has also been hypothesized that DMP1 may directly function as a transcription factor, however, recent data have shown that targeted nuclear expression of DMP1 in osteocytes and osteoblasts did not rescue the dental phenotype of DMP1 null mice (Lin et al., 2014). Therefore the mechanism responsible for DMP1 regulation of FGF23 is still not completely known.

While the bone phenotype of ARHR has been investigated, very little is known about other organ systems that may also be altered in ARHR or other genetic diseases of hypophosphatemic rickets. In addition to bone abnormalities, families with ARHR have also been reported to have nerve deafness, learning disabilities, and dental abnormalities (Farrow et al., 2009; Makitie et al., 2010). Our research group has been very interested in the relationship between bone and muscle health and so we specifically were interested in the skeletal, cardiac, and vascular smooth muscle function of this condition. It has previously been shown that XLH patients display muscle weakness and the Hyp mouse model of XLH displays reduced strength (Aono et al., 2011; Veilleux et al., 2013). Therefore, we wanted to determine if the *Dmp1* null mouse which closely mimics the human ARHR phenotype with abnormal dentinogenesis, chondrogeneisis, and mineralization (Ye et al., 2004, 2005; Ling et al., 2005; Feng et al., 2006) also has muscle weakness. These findings would potentially yield insights into common mechanisms for skeletal muscle phenotypes between XLH and ARHR. For our study, *Dmp1* null and wild-type (WT) mice were sacrificed and a fast-twitch, glycolytic muscle (extensor digitorum longus muscle–EDL), a slow-twitch oxidative muscle (soleus muscle–SOL), the heart and aorta were removed and the contractile responses to various challenges were studied. Importantly, a muscle phenotype was identified which may have implications for ARHR patient health.

## Materials and methods

### Animals

Twenty-two to 30 week-old wild type (WT) and D*mp1* null mice of both sexes were used in experiments. The Dmp1 null mice were generated with exon 6 deletion as previously described (Feng et al., 2003) and crossed on a C57BL/6 and 129 Sv mixed background. All mice were housed in a temperature-controlled (22 ± 2°C) room with a 12-h:12-h light/dark cycle. Animals were fed *ad libitum*. This study was carried out in accordance with the recommendations and approval of the Institutional Animal Care and Use Committee of the University of Missouri-Kansas City.

### Genotyping

Mice were genotyped using DNA isolated from tail segments obtained at weaning. Tail segments were digested in 10 mM Tris-HCl pH 8.0 containing 25 mM EDTA, 0.5% SDS, 50 mM NaCl, and 0.5 mg/ml proteinase K. A saturated solution of sodium chloride was then added to the digests to precipitate residual contaminating protein. Extracts were centrifuged for 25 min at room temperature and the DNA containing supernatant was precipitated using 80% isopropanol. Resulting pellets were then washed with 70% ethanol and allowed to air dry. Dried pellets were hydrated with 0.01 M Tris-HCl buffer (pH 8.0) containing 1 mM EDTA, and, the DNA concentration was determined by UV spectrophotometry.

DNA samples were genotyped using PCR with gene specific primers for *Dmp1* [forward 5′-GAGTGCGAT CTTCCTGAGGCCGATACTGTC-3′ and reverse 5′CGCGG CTGAAATCATCATTAAAGCGAGTGG-3′] and WT [forward 5′-GCCCCTGGACACTGACCATAGC-3′ and reverse 5′-CTG TTCCTCACTCTCACTGTCC-3′]. PCR products were evaluated by electrophoresis on 2.1% agarose gels followed by staining with ethidium bromide, and digitally imaged with a Fuji LAS4000 CCD scanner.

### Tissue extraction

Prior to use, mice were weighed and treated with heparin (5000 units/kg bodyweight) by intraperitoneal injection 15 min prior to sacrifice by cervical dislocation. Mouse hearts were quickly excised and placed into oxygenated Ringer's solution where residual blood was washed out and the atria were removed. The thoracic aorta was excised, placed in Hank's Buffered Salt Solution (HBSS, Invitrogen, Carlsbad, CA), and blood, fat, and excess connective tissues were carefully removed. As soon as the heart and aorta were extracted, intact EDL, and SOL muscles from the same mice were removed and placed in a dissecting dish containing oxygenated Ringer's solution where residual adipose and connective tissue were then removed.

### Skeletal muscle functional studies

Isometric force data was collected for EDL and SOL muscles (4 male /3 female mice) using an eight chambered system as we have previously described (Thornton et al., 2011; Park et al., 2012). Muscles bath experiments were run at room temperature, gassed with 100% oxygen, and bathed in Ringer's solution (142 mM NaCl_2_, 5 mM KCl, 1.8 mM MgCl_2_, 10 mM HEPES, 2.5 mM CaCl_2_, 10 mM glucose, pH 7.4). Data were collected and analyzed with PowerLab Software (AD Instruments; Colorado Springs, CO) customized for these experiments. Muscles were first equilibrated for 20 min to mimic conditions of normal activity (low duty cycle, ~1%). They were then subjected to the length-force relationship test to determine optimal length at which maximal force is achieved. Muscles were stimulated with a train of stimulation of 500 ms of frequencies ranging from 1 to 130 Hz at 1 ms pulses to generate force vs. frequency (FF) relationships. From the FF, the frequency producing maximal tetanic force (T_max_) was then used for the rest of the experiments. Muscles were stimulated every minute with (T_max_) for 5–10 min to assure that the preparation was stable. At the end of this period, muscles were treated with 5 mM caffeine that effectively releases calcium from the sarcoplasmic reticulum (SR) to test whether excitation-contraction coupling (ECC) was defective at the end of the recovery process. After *ex-vivo* contractility protocols, muscle dimensions, and masses were measured for determination of the normalized force corrected for physiological cross-sectional area (PCSA) based on the following equation: Force (N/cm^2^) = [force (g) × 0.00981 (m/s^2^) × muscle length (cm) × muscle density (1.06 g/cm^3^)]/[muscle weight (g)].

### Cardiac contractility measurements

Small heart clips were attached to the proximal septum and distal apex (Harvard apparatus; Holliston, MA) and then attached to a force transducer using silk thread running from the clips to the transducer as we have previously described (Touchberry et al., 2010). The intact hearts (7 male/3 female mice) were hung vertically between bipolar platinum stimulating electrodes suspended in 25-mL glass tissue chambers (AD Instruments, Colorado Springs, CO) superfused with Ringer's solution (NaCl 140 mM, CaCl_2_ 2.5 mM, KCl 2.0 mM, K_2_HPO_4_ 1.5 mM, MgSO_4_ 1 mM, HEPES 10 mM, pH 7.4) continuously bubbled with 100% O_2_ at room temperature. Muscles were rinsed three times and allowed to stabilize for 30 min in these conditions prior to experimentation. Maximum force of contraction was determined at 10 V, 5 ms duration, and 1 Hz frequency (Grass stimulator SD 9; Quincy, MA) and hearts were stretched to the length of maximal force development. The contractile data were recorded and analyzed on Lab Chart 6 software; AD Instruments (Colorado Springs, CO). The isometric tension during maximal contraction of *Dmp1* null and WT was normalized to heart weight and is presented as mN/g. Waveform analysis was also conducted for slope (mN/s), and the rate of relaxation, or tau, during the period of maximal contraction for both *Dmp1* null and WT.

### Cardiac gene expression measurements

Total RNA was isolated from *Dmp1* null and WT mouse ventricles using the Total RNA isolation kits from IBI Scientific (Peosta, IA). One step real-time RT-PCR was performed as previously described (Wacker and Godard, 2005) using the TaqMan RNA-to-CT 1 step kit from Applied Biosystems (Carlsbad, CA). Premade TaqMan primer/probes for genes of interest as well as the housekeeping gene, β-actin, were ordered from Applied Biosystems. Genes involved in cardiac hypertrophy or heart failure which included atrial naturietic peptide (ANP, Mm01255747_g1), skeletal muscle α actin (SkAct, Mm00808218_g1), β-myosin heavy chain (βMHC, Mm00600555_m1), α-myosin heavy chain (αMHC, Mm00440359_m1), sarcoplasmic endoplasmic reticulum calcium ATPase (SERCA2a2, Mm01201431_m1) as well as genes involved in cardiac remodeling, vimentin (Mm01333430_m1), and collagen 1a1 (Mm00801666_g1), were tested. Relative gene expression was quantified by 2^−ΔCT^ calculations utilizing β-actin as the housekeeping gene. Fold change was then calculated by dividing *Dmp1* null by WT expression and the results were averaged. One fold change equal expression to the WT and is indicated by a dashed line in **Figure 4**. β-actin was chosen as a housekeeping gene as *Dmp1* null and WT had minimal differences in β-actin gene expression.

### Aortic isometric tension myography

Aortic segments 3–4 mm in length (8 male/5 female mice) were mounted on pins in chambers of a DMT 610M wire myograph system (Danish Myo Technology A/S, Aarhus N, Denmark) containing Krebs buffer (in mM: 119 NaCl, 4.7 KCl, 0.24 NaHCO_3_, 1.18 KH_2_PO_4_, 1.19 MgSO_4_, 5.5 glucose, and 1.6 CaCl_2_) saturated at 37°C with a gas mixture containing 20% O_2_/5% CO_2_/75% N_2_ (Airgas Mid-South Inc., Tulsa, OK) at 37°C. Aortic rings were progressively stretched to 0.75 g equivalent force passive tension in 0.1 g steps and allowed to equilibrate for 45 min as described previously (Silswal et al., 2011, 2012). Force changes were recorded using an ADinstruments PowerLab 4/30 and associated LabChart Pro software (v6.1) running on a standard Windows XP computer platform. To assess the quality of the preparation before performing experiments, aortic rings were exposed to isotonic KCl (40 and 80 mM) and a concentration response curve to prostaglandin F_2α_ (PGF_2α_; 1 nM-10 μM) or 5-HT (1 nM-10 μM) was performed. The contraction response was followed by a concentration response curve to acetylcholine (ACh; 1 nM-10 μM) to evaluate endothelial-mediated relaxation. Similarly a relaxation response curve was measured after the nitric oxide donor, sodium nitroprusside (1 nM-10 μM) was carried out after precontraction with PGF_2α_ to assess smooth muscle relaxation. Contraction data was presented as g of tension and relaxation was presented as a percent relaxation compared to the maximal contraction produced by PGF_2α_ or 5-HT.

### Statistical analysis

All graphs were made and statistical procedures were performed using GraphPad Prism 6.0 or Origin 6.1. Data are presented as means ± S.E.M. Skeletal and cardiac muscle contractility data were compared using a paired *t*-test. Skeletal muscle sample numbers included muscles from the right and left limbs of the same animal. These muscles were averaged to yield one independent sample. Cardiac muscle gene expression analysis was compared using a one-way analysis of variance followed by a Tukey *Post-hoc* adjustment. Aortic contraction and relaxation experiments were analyzed using a two-way analysis of variance. Significance levels were set at *p* < *0.05* for experiments.

## Results

### Skeletal muscle

**Figure 1:**
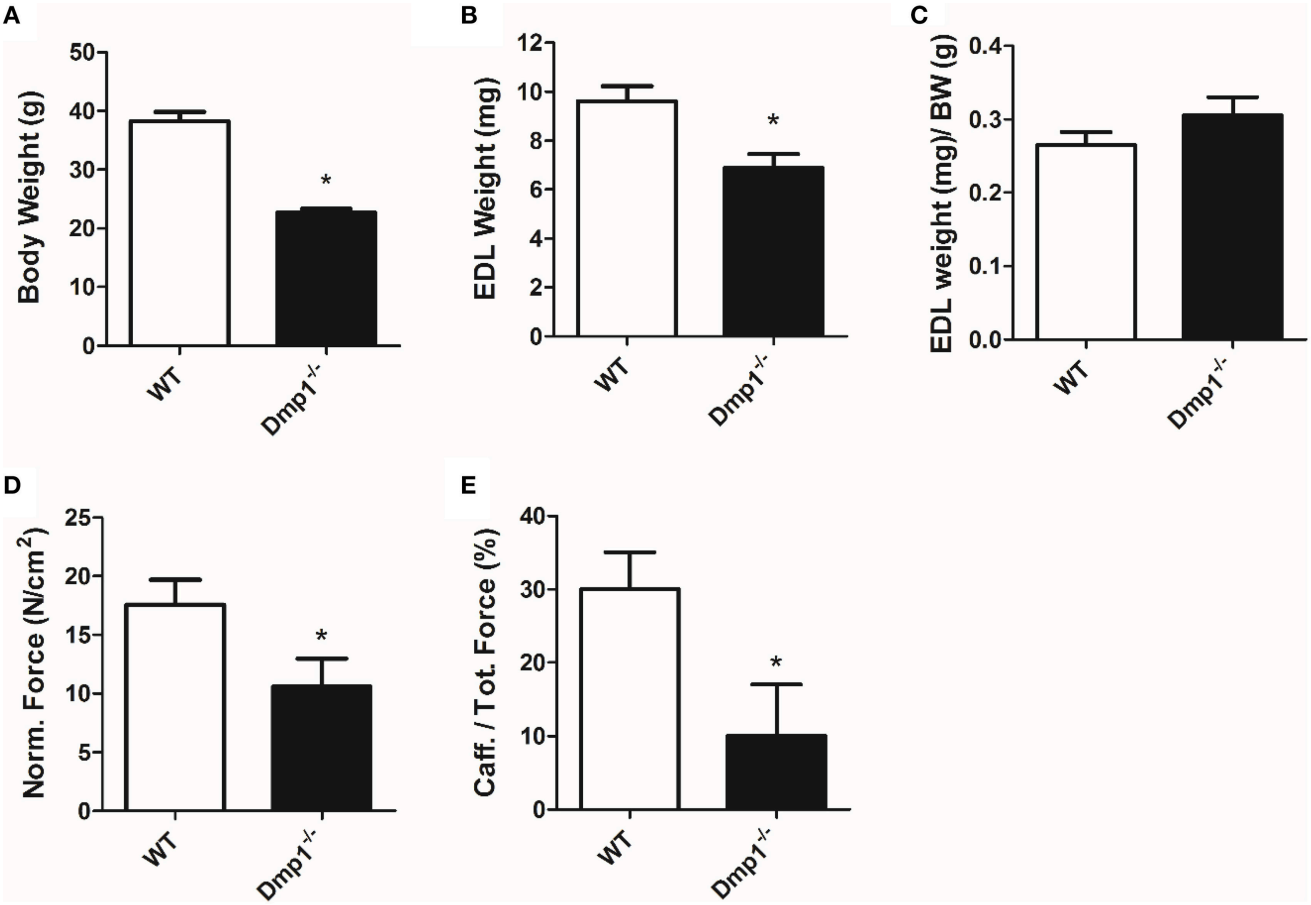
***Dmp1* null mice are smaller and the EDL muscles of *Dmp1* null are weaker than WT. (A)** Average body weights of WT and *Dmp1* null. **(B)** Average values of the dry blotted wet weights of EDL muscles from WT and *Dmp1* null mice illustrate the decreased mass in EDL from the null mice. **(C)** However, there was no difference in EDL weights once normalized to body weight (BW). **(D)** The physiological cross sectional area normalized force of EDL muscles from the *Dmp1* null is significantly lower than that produced by the EDL muscles from the WT mice suggesting that there may be additional factors beyond atrophy that contribute to muscle weakness. **(E)** Average caffeine-induced contractions in EDL muscles from *Dmp1* null show that muscles from the null mice produce significantly lower levels of force in response to SR calcium release induced by caffeine. These data reinforce the hypothesis that factors such as problems in ECC may account for the loss of force in the EDL. Data are mean ± SE; *n* = 7 (4 male/3 female); ^*^indicates statistical significance compared to WT (*P* < 0.05).

**Figure 2:**
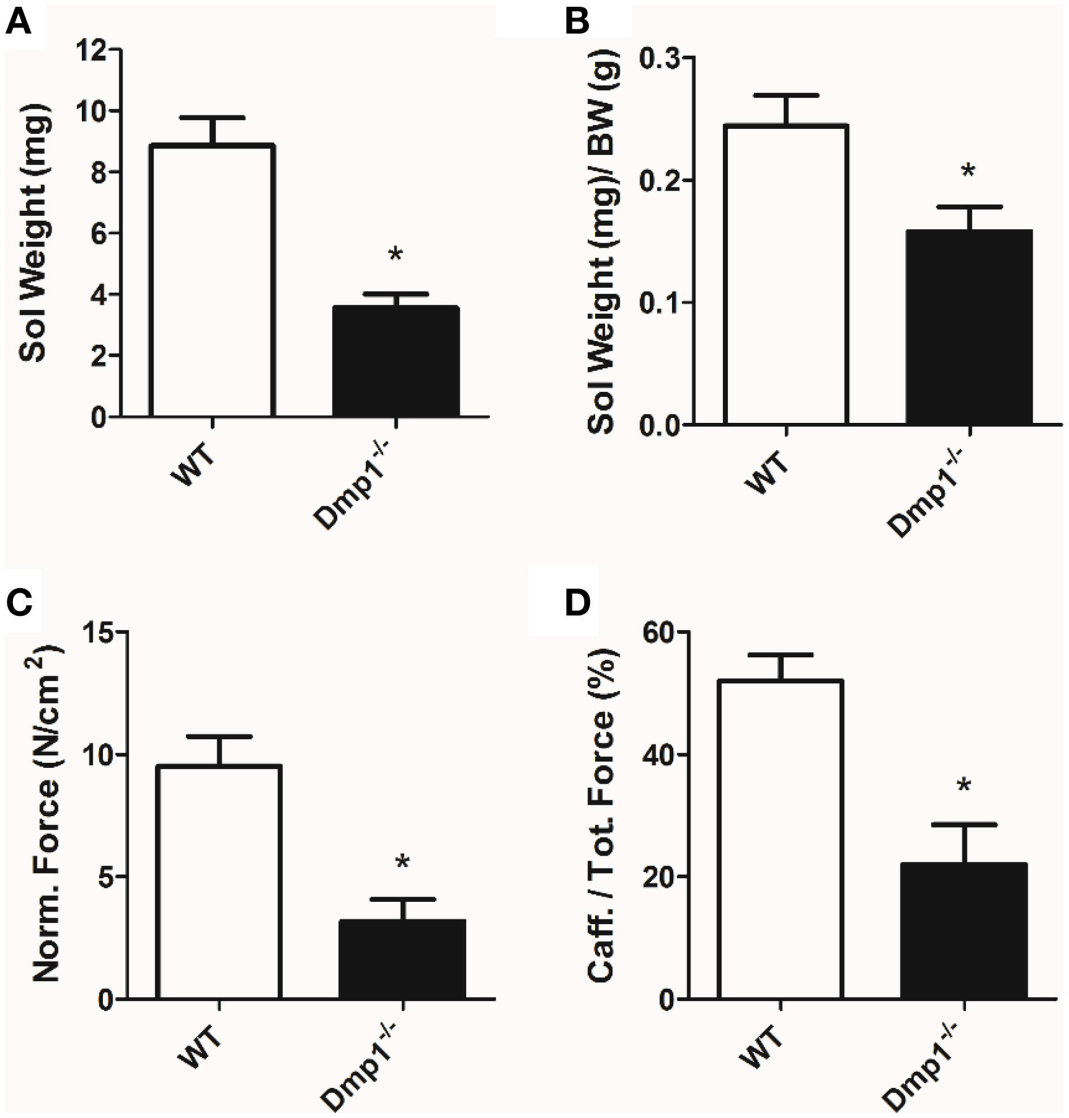
**SOL muscles from *Dmp1* null are smaller and weaker than WT. (A)** Average values of the dry blotted wet weights of SOL muscles from WT and *Dmp1* null mice illustrate decreased mass in SOL from the null mice. **(B)** The SOL muscles were still smaller than WT even after normalizing for body weight (BW). **(C)** The physiological cross sectional area normalized force of SOL muscles from the *Dmp1* null mice is reduced by 2/3 when compared to SOL muscles from WT mice suggesting that there may be additional factors beyond atrophy that contribute to muscle weakness. **(D)** Average caffeine induced contractions in SOL muscles from *Dmp1* null show that muscles from the null mice produce significantly lower levels of force in response to SR calcium release induced by caffeine. These data reinforce the hypothesis that factors such as problems in ECC may account for some of the loss of force in the SOL. Data are mean ± SE; *n* = 7 (4 male/3 female); ^*^indicates statistical significance compared to WT (*P* < 0.05).

In general, we analyzed data for gender differences, but found no difference between male or female animals in skeletal, cardiac, or vascular smooth muscle responses, therefore genders were combined for final analysis in the three muscle types. For skeletal muscle, we found that the *Dmp1* null animals and skeletal muscles were smaller than WT (Figures 1A,B, 2A; *P* < 0.05). While there was no difference in the normalized weight of the EDL, the SOL was smaller than WT after correcting for BW (Figure 1C, *P* > 0.05; Figure 2B, *P* < 0.05). The absolute force of both EDL and SOL of *Dmp1* null mice was reduced as compared to WT mice (Figures 1B, 2B; *P* < 0.05). After normalizing the forces of all muscles to the PCSA (which takes into account not only the mass, but also the length and the density of the muscles), the normalized force was reduced in both EDL and SOL (Figures 1D, 2C, *P* < 0.05). These data indicate, that size alone did not account for the reduced force of contraction. Skeletal muscles depend heavily on intracellular calcium stores in the sarcoplasmic reticulum (SR) to release calcium during ECC. To test the possibility that *Dmp1* null muscles are weaker due to alterations in SR calcium handling, we treated each of these muscles with 5 mM caffeine, an agent that effectively releases calcium from the SR. Interestingly, we found that both EDL and SOL muscles had reduced responses to caffeine, suggesting defects in the ECC that are responsible for the muscle weakness (Figures 1E, 2D; *P* < 0.05).

### Cardiac muscle

**Figure 3:**
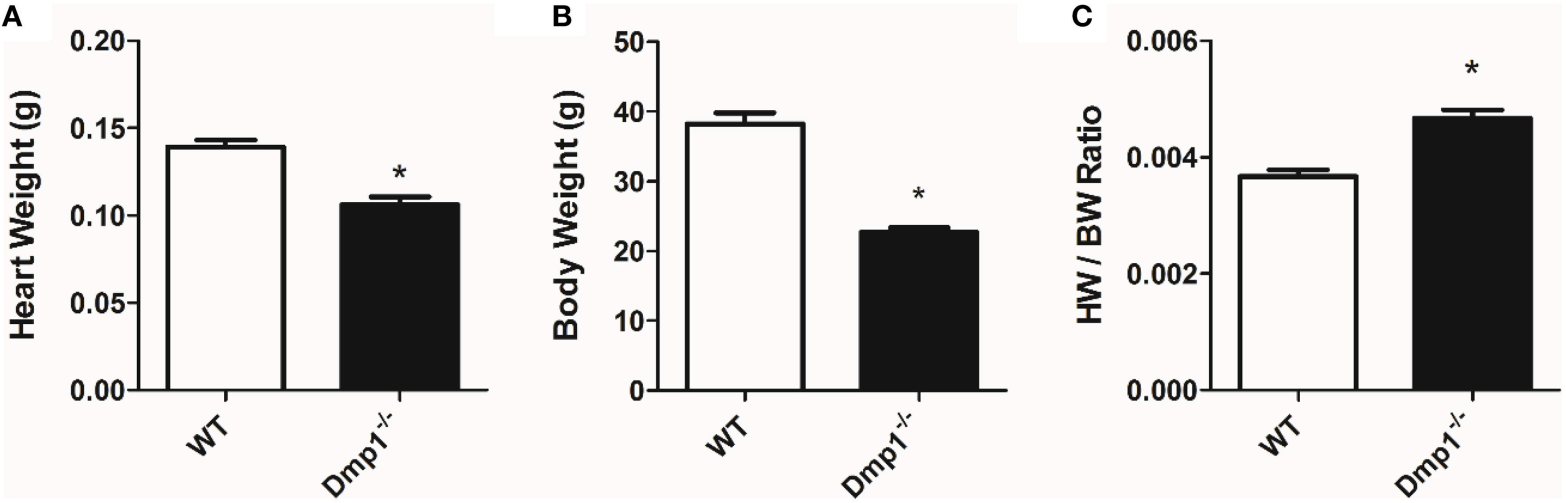
***Dmp1* null mice have altered heart weight to body weight ratios**. *Dmp1* null mice showed reduced heart weights **(A)** and body weights **(B)**. **(C)** While both heart weight and body weights were reduced, heart weight to body weight ratio was higher in the *Dmp1* null mice compared to WT mice. Data are mean ± SE; *n* = 10 (7 male/3 female); ^*^indicates statistical significance compared to WT (*P* < 0.05).

*Dmp1* null mice had a significantly reduced body mass (−40.1%) and heart weight (−23.8%) when compared to WT mice (Figures 3A,B; *P* < 0.05). However, the proportion of HW size to BW size (the HW/BW ratio) was significantly increased (27.3%) in the *Dmp1* null mice compared to WT (Figure 3C; *P* < 0.05).

**Figure 4:**
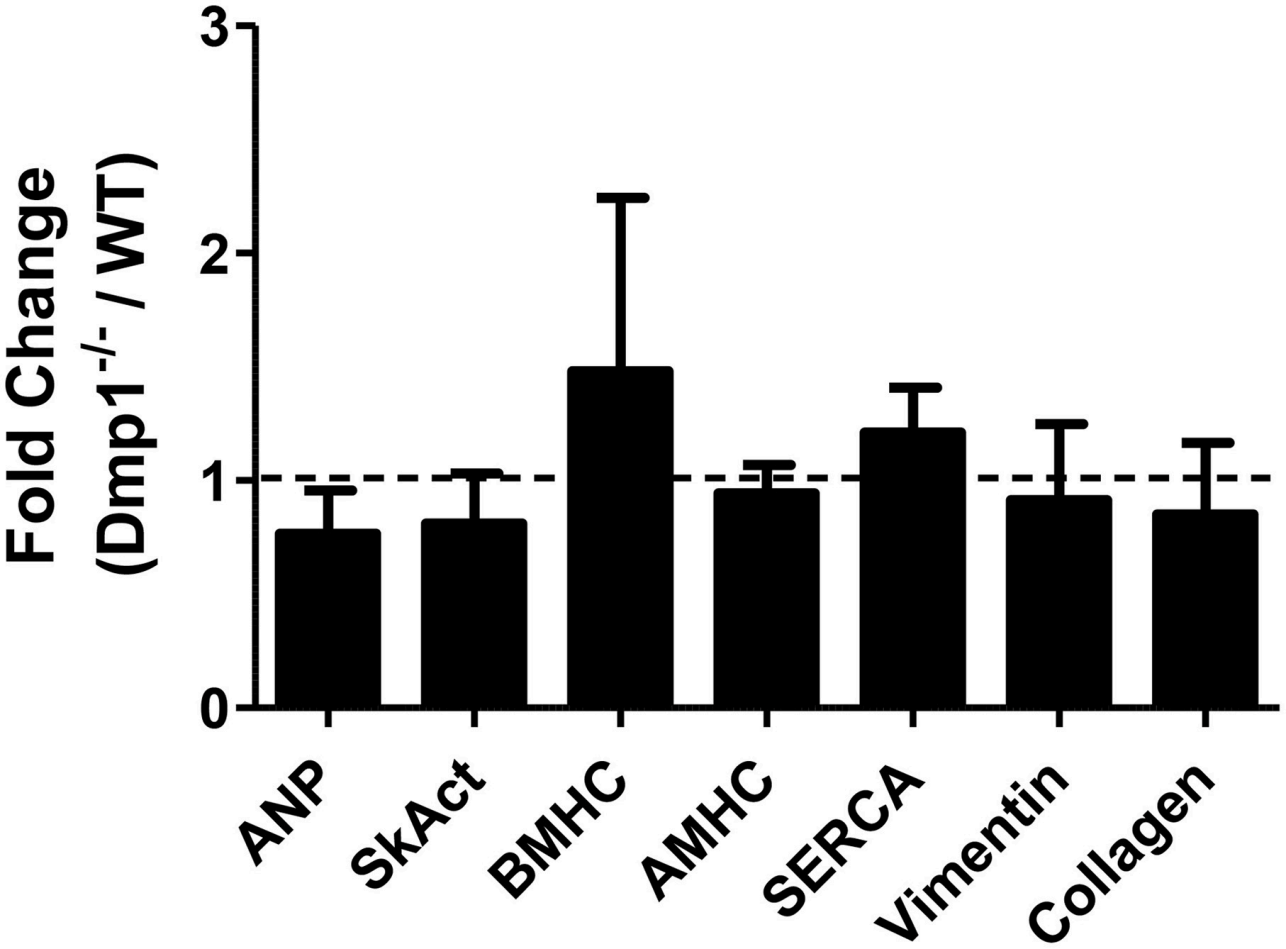
**No increase in genes involved in hypertrophic signaling or remodeling in the *Dmp1* null mice**. To dermine if the increase in heart weight to body weight ratio in *Dmp1* null mice was due to pathological cardiac remodeling, we determined the expression of hypertrophy-associated genes atrial natriuretic peptide (ANP), skeletal muscle α-actin (SkAct), β-myosin heavy chain (βMHC), and α-myosin heavy chain. *Dmp1* null mice did not show any significant elevations in these markers of hypertrophy. *Dmp1* null mice did not exhibit any changes in the expression of sarcoplasmic endoplasmic reticulum calcium ATPase (SERCA), which is known to be downregulated in heart failure. Lastly, *Dmp1* null mice did not show an increased expression of vimentin (marker of cardiac fibroblasts) or collagen 1a1 (marker of fibrosis). Data are mean ± SE; *n* = 5 (male); *P* > 0.05.

Since we observed an increase in relative heart size in *Dmp1* null mice, and increases in HW/BW ratios can be indicative of pathological hypertrophy, we analyzed ventricular heart tissue for genetic markers of pathological hypertrophy and remodeling as well as changes in contractility. We did not observe an increase in genes associated with pathological cardiac hypertrophy (ANP, Sk. Act., β-MHC) or genes associated with fibroblasts (vimentin) or remodeling (collagen) (Figure 4; *P* > 0.05) in *Dmp1* null mice compared to WT mice. These data suggest that the proportionately larger hearts from *Dmp1* null did not show classical markers of pathological hypertrophy.

**Figure 5:**
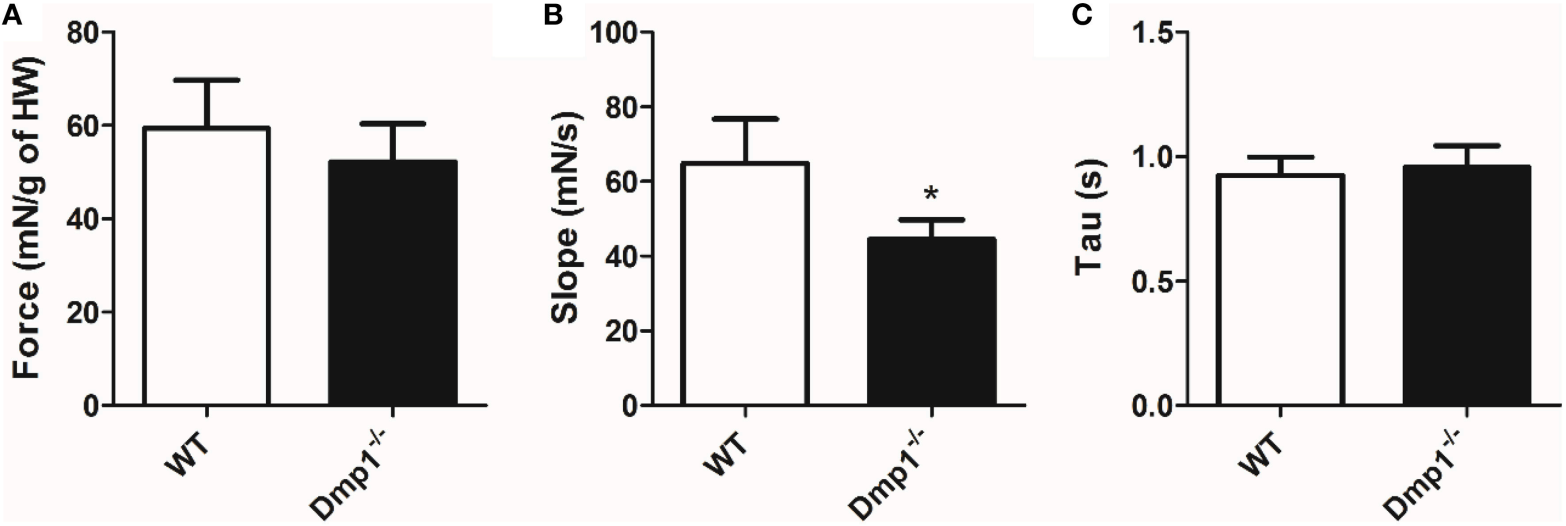
**Comparison of cardiac muscle contractile waveforms between WT and *Dmp1* null. (A)** There were no differences in isometric force production corrected for heart weight between WT and *Dmp1* null mice. **(B)** *Dmp1* null mice did present with a reduced slope, suggest a reduction in the rate at which isometric tension is developed. **(C)** There were no differences in tau (rate of relaxation), between WT and *Dmp1* null mice, suggesting calcium reuptake is unaltered in *Dmp1* null mice. Data are mean ± SE; *n* = 10 (7 male/3 female); ^*^indicates statistical significance compared to WT (*P* < 0.05).

Next, we tested *ex-vivo* whole heart contractility using an isolated organ bath with stimuli that produced maximal contractions. Maximal tension (adjusted for HW) at 1 Hz stimulation was not statistically different between WT and *Dmp1* null mice hearts (Figure 5A; *P* > 0.05). However, there was a decrease in the maximum slope of the contractile waveform indicating that the maximum rate of force development was reduced in the *Dmp1* null mice hearts compared to WT (Figure 5B; *P* > 0.05). There was no difference in the rate of relaxation (Tau) between hearts (Figure 5C; *P* > 0.05). These data show that the *Dmp1* null hearts were able to contract to the same extent per g of heart weight, but the rate of force development was reduced. Overall, the contractile data support the gene expression data indicating that there was no overt cardiac pathology at the time-point studied since there was little dysfunction in maximal contraction.

### Vascular smooth muscle

**Figure 6:**
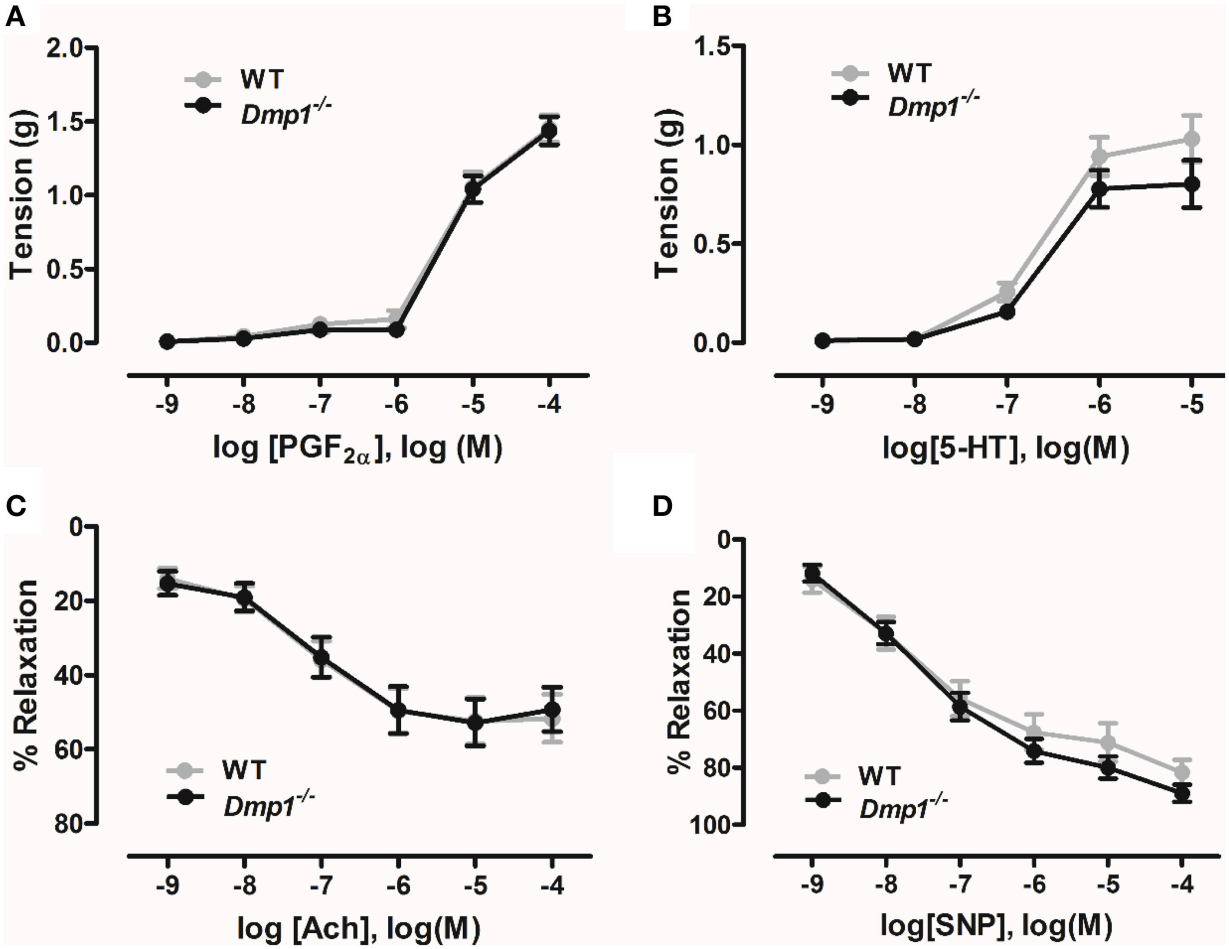
***Dmp1* null mice do not display impaired aortic contractions or relaxation**. Contractile response curves to prostaglandin F_2α_ (PGF_2α_) **(A)** and 5-hydroxytryptamine (5-HT) **(B)** were not different between WT and *Dmp1* null mice suggesting that smooth muscle function was normal. Relaxation response curves to acetylcholine (ACh) **(C)** and the NO donor, sodium nitroprusside (SNP) **(D)** on PGF_2α_-precontracted aortic rings from WT and *Dmp1*^−∕−^mice were also not different suggesting there was not impairment in endothelial-dependent or endothelial-independent smooth muscle relaxation. Data are mean ± SE; *n* = 13 (8 male/5 female), *P* > 0.05.

Isometric tension myography was conducted on isolated aortic rings to determine if there were differences in contraction or relaxation of the vasculature in the *Dmp1* null mice compared to WT. No statistical differences were observed in contraction responses to increasing concentrations of either PGF_2α_ or serotonin comparing WT and *Dmp1* null (Figures 6A,B; *P* > 0.05). When vessels were precontracted with PGF_2α_, there was no difference in relaxation produced by increasing concentrations of ACh indicating that there was no impairment of aortic endothelium in *Dmp1* null mice (Figure 6C; *P* > 0.05). Likewise, the nitric oxide donor (endothelium-independent relaxation) induced a similar relaxation response between WT and *Dmp1* null (Figure 6D; *P* > 0.05). Collectively, these results demonstrate there was no impairment in vascular smooth muscle or endothelium of aorta in *Dmp1* null mouse compared to the WT.

## Discussion

ARHR patients are known to display rickets/osteomalacia, but little is known about skeletal muscle or cardiovascular function in ARHR or other heritable diseases of hypophosphatemia. In this study, for the first time, the function of skeletal and cardiac muscle as well as vascular smooth muscle function in the *Dmp1* null mouse model of ARHR was analyzed. Similar to ARHR patients, these mice display deformed bones and growth plates, shorter and wider long bones, bony protrusions, delayed blood vessel invasion of bone, altered canaliculi, impaired fluid flow through the canaliculi, less organized osteocytes, and a decreased mineral to matix ratio (Ling et al., 2005; Rios et al., 2005; Feng et al., 2006). Understanding the muscle function in this condition has great significance for patients suffering from rickets/osteomalacia.

### Skeletal muscle phenotype

We found that skeletal muscle force in both a fast-twitch and a slow-twitch muscle were reduced. The *Dmp1 null* mice and muscles were smaller than WT, but even after correcting for the cross-sectional area of the muscle, we found that the *Dmp1 null* EDL and SOL muscles were significantly weaker indicating that atrophy alone cannot account for the reduction in force production. Since this phenomenon was observed in both the EDL and SOL, the mechanism of impairment likely involved general mechanisms of skeletal muscle contraction and was not due to specific fiber type differences. Both muscle types had reduced tetanic force in response to caffeine administration compared to WT demonstrating that the calcium release mechanism during ECC was impaired. Some possibilities for this impaired ECC could be reductions in calcium levels in the SR, problems in dihydropyridine receptor (DHPR) interaction with the ryanodine receptor (RyR), or alterations in proteins (and/or their phosphorylation states) such as SERCA, the calcium binding protein calsequestrin, RyR1, or myosin heavy chain. These all are important future targets to analyze for both human as well as animal studies involving genetic hypophosphatemic diseases such as ARHR.

Patients suffering from osteomalacia/rickets have reported muscle deficits (Schott and Wills, 1976; Veilleux et al., 2012). To our knowledge, there have been no detailed studies specifically documenting muscle function in ARHR patients, but it has been found that hypophosphatemic rickets patients have reduced function in lower limb muscle function tests (Veilleux et al., 2012). Similarly, calf muscle force, but not cross-sectional area, was lower in X-linked hypophosphatemia (XLH) patients compared to controls, while the distal tibia had an increased bone cross sectional area, but lower cortical bone mineral density (Veilleux et al., 2013). In addition, there have been skeletal muscle studies in rodent models of XLH (using the Hyp mouse). XLH results from mutations in PHEX which then allows FGF23 to go unregulated and reduces phosphate levels comparable to ARHR. The Hyp mouse model of XLH rickets has reduced grip strength as well as reduced spontaneous movement (Aono et al., 2011). Our data in the ARHR model would thus seem to have consistency to the findings in XLH and suggest a shared cause.

The underlying direct mechanism causing the changes in muscle function we observed still needs to be elucidated. It is possible that the changes in plasma ion concentrations in the *Dmp1* null are the direct cause for the muscle phenotype. Previous reports from our laboratory and others have measured serum concentrations of various ions and hormones in the blood at the same approximate age as the mice in this study. The average values from these studies are as follows: FGF23 = 1169 ± 158 pg/ml; Pi = 4.4 ± 0.6 mg/dl; Ca^2+^ = 7.9 ± 0.4 mg/dl; 1,25 Vit. D = 65.2 ± 27 (Feng et al., 2006; Liu et al., 2008; Lu et al., 2011). Changes in phosphate, calcium, or Vitamin D could play a role in altering the function of the skeletal muscle, although reduced phosphate alone was the primary mechanism for causing weakness in slow-twitch muscle fibers in rats with severe vitamin D deficiency (and hypocalcemia) (Schubert and Deluca, 2010). The potential mechanisms for hypophosphatemic muscle weakness are still largely unresolved, but likely involve the metabolism of ATP, phosphorylation of myosin and actin filaments, alteration in ion pumps and calcium handling, or changes in mitochondrial function (Fuller et al., 1976; Brautbar et al., 1983; Hettleman et al., 1983; Johnson et al., 1989). However, an important point to note is that the muscles in our experiments were placed in a Ringer's solution with normal phosphate and calcium for each muscle bath experiment and had time to equilibrate after removal from the animal. Furthermore, Veilleux et al. found no significant influence of serum phosphorus, PTH, or treatment status on the muscle weakness identified in their hypophosphatemic patients (Veilleux et al., 2012). Nevertheless, it is possible that the chronic exposure to low phosphate may have remodeled the muscles to work with lower force or at lower phosphate levels.

Changes in bone structure as well as inactivity may also induce the skeletal muscle phenotype in the *Dmp1* null. In hypophosphatemic patients with muscle function deficits, it was observed that patients without lower limb deformities had better muscle function than those with severe deformities (Veilleux et al., 2012). In the case of the *Dmp1* null, there is likely more to the mechanism than simply muscle disuse, as atrophy alone could not account for the muscle weakness in our studies; however, it remains to be determined if muscles are, in part, adapting to altered bone. This hypothesis may not be able to explain the findings in our organ bath system as the muscles were tested independently of bone attachment and at optimal stretch.

It is also possible that the altered bone phenotype in the *Dmp1* null may have impacted normal bone-muscle crosstalk through paracrine and endocrine agents (Isaacson and Brotto, 2014). Our research group has been interested in the crosstalk between bone and muscle and the role of bone health in directly contributing to skeletal muscle and cardiovascular health. While it is well-accepted that muscle contraction can increase mechanical forces on bone and lead to altered bone density, it is becoming more commonly accepted that bone can act in an endocrine manner to communicate with organs such as the kidney, brain, and muscle to alter systemic physiological functions (Brotto and Johnson, 2014). As far as bone-muscle interactions, Gorski et al. (2015) have recently shown that conditional deletion of *Mbtps1* protease in bone results in increased mass, contraction force, and increased regenerative capacity in SOL muscles of 10–12, but not 3 month-old mice. There were no changes in the MBTPS1 protease expression observed in SOL muscles and it was concluded that altered bone-muscle crosstalk was involved in producing the changes in muscle phenotype. Specifically, bone can secrete factors in a paracrine manner which has important implications for local biochemical crosstalk between bone and skeletal muscle. Using fluorescent tracers, the periosteum has been shown to be permeable to molecules under 40 kDa such as PGE2, IGF-1, IL-15, and FGF-2 (Lai et al., 2014). Our research group has shown that PGE2 is an important factor in MLO-Y4 osteocyte-like conditioned media which accounts for changes in myoblast growth, differentiation, and proliferation (Mo et al., 2012, 2015). Additionally, another group has shown that bone morphogenic proteins are important regulators of skeletal muscle mass (Sartori and Sandri, 2015). Beyond paracrine factors, bone cells such as osteocytes and osteoblasts are also well-known to secrete various hormones such as FGF23, osteocalcin, and sclerostin that can target multiple organs such as the kidney, parathyroid, brain, and adipose. FGF23 in particular has received special attention recently due to its ability to directly influence the heart and vasculature (Faul et al., 2011; Touchberry et al., 2013; Silswal et al., 2014). Interestingly, treatment with anti-FGF23 antibody therapy was able to improve muscle weakness and the reduced activity in the Hyp mouse (Aono et al., 2011). However, it remains to be determined if FGF23 has a direct action on skeletal muscle.

Lastly, it is possible that deletion of *Dmp1* directly altered the muscle phenotype. This is likely less probable as DMP1 has been shown to be expressed primarily in bone cells such as osteocytes and osteoblasts. There is a report demonstrating that DMP1 is also expressed in some soft tissues such as the brain, pancreas, kidney, and liver, with highest expression in the brain and pancreas (Terasawa et al., 2004) and a report demonstrating DMP1 expression in coronary arteries (Van Venrooij et al., 2014). Terasawa et al. (2004), showed low gene expression in skeletal muscle tissue, however, it was not clear what cell type may be responsible. To our knowledge there have been no reports demonstrating expression in cardiac myocytes. We hypothesize that the global deletion of *Dmp1* is most likely not responsible for the phenotype as other animal models of reduced bone formation or hypophosphatemia such as Hyp mice also display muscle weakness. In the future it will be interesting to determine if the Hyp mouse and *Dmp1* null mouse have similar specific molecular mechanisms inducing the phenotype.

### Cardiovascular effects

While there have been clinical studies linking lower bone mass with a greater risk of heart failure (Pfister et al., 2014), there have been very few studies analyzing cardiovascular function in ARHR or specifically studying cardiac muscle function in bone disease. One study with young XLH subjects found left ventricular hypertrophy in 7 of 13 patients (diagnosed by EKG or ultrasonography) and also an increase in diastolic blood pressure during an exercise stress test (Nehgme et al., 1997). Therefore, there was strong rationale to determine if changes in cardiac or vascular function occur in this ARHR model.

The hearts in the *Dmp1* null mice displayed an increase in HW/BW ratio indicative of hypertrophy. Gene expression analysis of markers for pathological hypertrophy or remodeling (fibrosis), however, were not elevated. There was also no difference in the total contractile force produced after correcting for the heart weight between WT and *Dmp1* null. These results would indicate that a pathological condition was not present at the time point studied. Interestingly, Nehgme et al. (1997) as well as Vered et al. (1990) found no changes in left ventricular ejection fraction in XLH patients despite cardiac hypertrophy being present. Further research remains to determine if our findings point toward an adaptation of physiological hypertrophy, a difference in growth and development between body and heart mass, or if a pathology would arise later in age of the mouse.

While the total force was not different, the rate of force development was reduced. Similar to our findings in skeletal muscle, this result may indicate some dysfunction in cardiac muscle ECC or an adaptation of the muscle to the conditions. These differences appear to only extend to excitation as the rate of relaxation was not altered. We paced these hearts at 1 Hz in order to reduce myocardial stress and oxygen demand on the heart, similar to standard protocols (Kirchhefer et al., 2004; Gonzalez-Munoz et al., 2008; Touchberry et al., 2010). Since there was a reduction in rate of force development, it is possible that hearts stressed at higher rates (reduced diastolic time) may show a more dramatic phenotype or cardiac weakness over time. It is also possible that cardiac muscle did not show as extreme a phenotype as skeletal muscle because of differences in ECC such as a larger reliance on extracellular vs. intracellular calcium in cardiac vs skeletal muscle.

Our group and others have documented that FGF23 can induce cardiac hypertrophy as well as alter calcium handling and contractility (Faul et al., 2011; Touchberry et al., 2013; Grabner et al., 2015). Work by Faul et al. has shown that exogenous FGF23 at 15,000–100,000 pg/ml produced hypertrophy *in vitro* as well as *ex-vivo* (Faul et al., 2011; Grabner et al., 2015). Since the levels of FGF23 in the *Dmp1* null mouse have been measured between ~900 and 1400 pg/ml at 5–6 months of age (Feng et al., 2006; Liu et al., 2008; Lu et al., 2011), it is possible that these hearts did not develop pathological hypertrophy because FGF23 did not reach high enough serum levels or that FGF23 at lower serum levels works in concert with other factors (such as those present in chronic kidney disease–CKD) to induce pathological hypertrophy.

There were no significant differences in vascular function in the *Dmp1* null aorta. Nehgme et al. in their study with XLH patients did observe increased diastolic blood pressure indicative of vascular dysfunction (Nehgme et al., 1997). They hypothesized that calcification of arterial walls may have altered vessel compliance and production of vasodilators resulting in increases in systemic vascular resistance and compensatory hypertrophy. Since we did not observe any endothelial impairment or dysfunction, it is possible that ARHR does not directly induce vascular dysfunction and that vascular changes may have been due to the treatments that XLH patients received. This is supported by research showing vascular dysfunction such as calcification tends to occur due to changes in high phosphate (Scialla et al., 2013). Interestingly, we have shown that treatment with 9000 pg/ml FGF23 can induce endothelial dysfunction in mouse aorta and have observed similar dysfunction in a mouse model of CKD (Silswal et al., 2014). Again, it is possible that the levels of FGF23 in the *Dmp1* null were not high enough to cause dysfunction or that additional factors present during CKD also play a role.

In summary, *Dmp1* null mice show dramatic skeletal muscle weakness, reductions in SOL size, increased HW/BW ratio, and reduced cardiac muscle rate of force development but not total force. Importantly, we have shown that in an ARHR mouse model, there are muscle phenotypes in addition to the skeletal phenotype. It will be critical that ARHR as well as other patients with hypophosphatemia and bone disease are checked for muscle function in addition to monitoring bone health.

## Author contributions

MW, MB, LBo, JA, CT, and NS helped to design experiments necessary for the study. LBr, CT, CE, NS, and MW collected the data. All authors helped to analyze and interpret the data. MW was the primary author of the manuscript and MB, CT, and NS made significant writing contributions. All authors edited, modified, and approved the final version of the manuscript.

## Funding

This work was supported by funding from National Institutes of Health grants 1-P01-AG039355-01A1 (LBo, MB, MW) and 1-RC2-AR-058962 (LBo, MB, MW, JA) and American Heart Association grants 11SDG5330016 (MW), SDG-0735053N (JA), and 11POST7650044 (NS).

### Conflict of interest statement

The authors declare that the research was conducted in the absence of any commercial or financial relationships that could be construed as a potential conflict of interest.

## Abbreviations

CKD: chronic kidney disease
DMP: dentin matrix acidic phosphoprotein
ARHR: autosomal recessive hypophosphatemic rickets
XLH: X-linked hypophosphatemia
EDL: extensor digitorum longus muscle
SOL: soleus muscle
FGF23: fibroblast growth factor 23
PCSA: physiological cross-sectional area
ECC: excitation contraction coupling.
